# Growth Hormone’s Effect on Adipose Tissue: Quality versus Quantity

**DOI:** 10.3390/ijms18081621

**Published:** 2017-07-26

**Authors:** Darlene E. Berryman, Edward O. List

**Affiliations:** 1 The Diabetes Institute at Ohio University, 108 Konneker Research Labs, Ohio University, Athens, OH 45701, USA; list@ohio.edu; 2 Edison Biotechnology Institute, 218 Konneker Research Labs, Ohio University, Athens, OH 45701, USA

**Keywords:** adipose tissue, obesity, growth hormone, growth factor-1 (IGF-1), bovine GH transgenic (bGH) mice, growth hormone receptor (GHR)-/- mice, GHR antagonist (GHA) mice, acromegaly, Laron syndrome, growth hormone deficiency

## Abstract

Obesity is an excessive accumulation or expansion of adipose tissue (AT) due to an increase in either the size and/or number of its characteristic cell type, the adipocyte. As one of the most significant public health problems of our time, obesity and its associated metabolic complications have demanded that attention be given to finding effective therapeutic options aimed at reducing adiposity or the metabolic dysfunction associated with its accumulation. Growth hormone (GH) has therapeutic potential due to its potent lipolytic effect and resultant ability to reduce AT mass while preserving lean body mass. However, AT and its resident adipocytes are significantly more dynamic and elaborate than once thought and require one not to use the reduction in absolute mass as a readout of efficacy alone. Paradoxically, therapies that reduce GH action may ultimately prove to be healthier, in part because GH also possesses potent anti-insulin activities along with concerns that GH may promote the growth of certain cancers. This review will briefly summarize some of the newer complexities of AT relevant to GH action and describe the current understanding of how GH influences this tissue using data from both humans and mice. We will conclude by considering the therapeutic use of GH or GH antagonists in obesity, as well as important gaps in knowledge regarding GH and AT.

## 1. Introduction

The GH/IGF-1 axis refers to the collective and coordinated actions of growth hormone (GH) and insulin-like growth factor-1 (IGF-1). GH, as its name would suggest, has anabolic effects on most tissues. Adipose tissue (AT) is an exception; GH, but not IGF-1, has a dramatic catabolic effect on AT, providing a significant source of the metabolic energy to support the growth of other tissues. Driven by the obesity epidemic, the last few decades have resulted in great progress in our understanding of AT, its alteration with the onset of obesity and its overall role in metabolic health. In turn, this newer research on AT has also refined our understanding of how GH alters this tissue, as well as provides additional insight as to the effects of altering GH action in the management of obesity.

## 2. Complexity of AT

### 2.1. Types of AT and Adipocytes

Adipocytes are the building blocks of AT and can occupy up to 90% of the tissue volume [1]. AT is generally classified as one of two discrete types: white AT (WAT) or brown AT (BAT). Despite being lumped under a single category due to their shared ability to store lipids, these tissues and their adipocytes differ in abundance, lineage, cellular composition and function. WAT, primarily composed of unilocular, lipid-laden white adipocytes, is present in far greater amounts than BAT, which is composed of multilocular brown adipocytes. WAT acts as the primary energy reserve in the body, storing excess nutrients as triacylglycerol (TAG) and releasing energy as free fatty acids (FFAs) and glycerol. BAT, with its greater mitochondrial content and high expression of uncoupling protein 1 (UCP-1), has a thermogenic role, dissipating excess energy through the production of heat. Although the importance of BAT in thermoregulation has been well understood for many years in newborns and many other species (including rodents and hibernating animals), recent advances in imaging technologies have allowed for the identification of BAT in adult humans [2]. In line with the proposed thermogenic functions of BAT, several clinical studies have reported seasonal variations in BAT activity, showing negative correlations with average outdoor temperatures [3,4,5]. This discovery along with the ability to modify BAT activity has fueled efforts to find novel therapies to activate BAT in order to accelerate weight loss and ameliorate associated diseases.

The more recent identification of beige, brite, or brown-like adipocytes, which are located within WAT depots, has further advanced our simplistic view of this tissue. These intermediary adipocytes are typically “paucilocular”, a designation that describes UCP-1-positive adipocytes with a lipid droplet size and distribution between that of white and brown. Like its brown counterpart, beige adipocytes have the capacity for thermogenesis, express UCP1 and can be activated in response to cold exposure or treatment with certain molecules [5]. The source of beige adipocytes is still debated although likely variable, as there is evidence for their formation from transdifferentiation of unilocular white adipocytes, from a unique precursor preadipocyte population, as well as select activation of other non-adipocyte cells within the tissue [6,7,8]. Recent evidence suggests the presence of functionally-distinct populations of beige adipocytes [9] that are molecularly distinct from brown and white adipocytes in both mice and humans [10,11].

While identifying differences among white, brown and beige adipocytes is of high interest, heterogeneity among white adipocytes has also been reported [12,13]. In fact, cell lineage tracing studies reveal a subpopulation of white adipocytes in visceral depots that is derived from the mesothelium [14], while the anterior subcutaneous and retroperitoneal WAT are derived from a distinct lineage most likely from a source outside the peritoneum. Importantly, this study shows intra-depot heterogeneity of the white adipocytes within visceral depots. There is also evidence of intrinsic heterogeneity of beige adipocytes even within a single WAT depot [15]. The physiological contribution of these adipocyte subpopulations and how they relate to adipocyte physiology and function is not yet known; however, understanding cellular ontogeny could have implications for understanding how AT is impacted by disease, hormones or therapeutic targets.

### 2.2. Endocrine Function

AT is now recognized as an endocrine organ. That is, WAT secretes a variety of potent hormones and cytokines, also known as “adipokines”, that have autocrine, paracrine and endocrine effects. Two of the best-studied adipokines are leptin and adiponectin, which were discovered almost simultaneously (leptin in 1994 and adiponectin in 1995) and revolutionized AT research. Leptin is secreted from adipocytes in response to food intake and is positively correlated with total fat mass. As a satiety hormone, leptin is known to bind to leptin receptors, primarily on the arcuate nucleus of the hypothalamus, to decrease food intake and increase energy expenditure. However, additional functions have been uncovered including the promotion of lipid oxidation, the modulation of innate and adaptive immune responses, as well as an influence on central nervous system diseases such as Alzheimer’s [16,17,18]. In contrast, adiponectin is negatively correlated with fat mass, is an anti-inflammatory and insulin-sensitizing hormone and can be used to predict insulin resistance and metabolic syndrome in overweight and obese patients [19,20]. Many other adipokines have been identified such as adipsin, fibroblast growth factor 21 (FGF21), apelin, chemerin, retinol binding protein-4, C-reactive protein, interleukin-6 (IL-6), resistin, tumor necrosis factor α and angiotensinogen [21]. Of note, some adipokines appear to be preferentially secreted by certain depots or specific cell types within adipose tissue. For example, both leptin and adiponectin are secreted in greater quantities by subcutaneous WAT compared to visceral [22,23]. Likewise, the adipokine omentin is mainly produced by the non-adipocyte cells (stromal vascular fraction (SVF)) of visceral depots [24,25], and visfatin is predominantly secreted by the macrophages of visceral depots [26]. Of note, BAT and beige adipocytes are also capable of adipokine secretion with some overlap with and some distinct secretion patterns to WAT [27]. For example, the “batokine” neuregulin 4 (NRG4), which targets the liver to inhibit de novo lipogenesis, appears to be preferentially secreted from BAT [28].

### 2.3. Depot Differences

**Figure 1:**
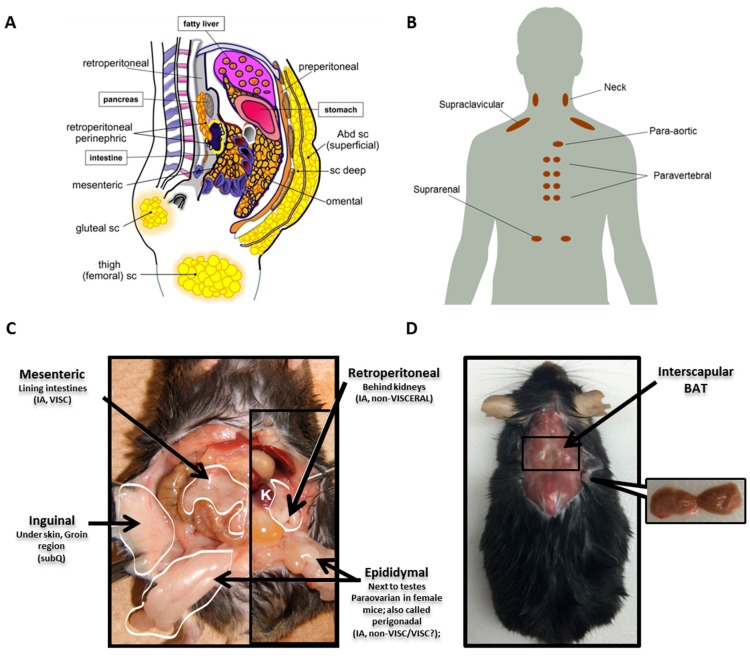
Distribution of white AT (WAT) and brown AT (BAT) in humans and mice. (**A**) Prominent human WAT depots are shown. Subcutaneous (subQ) depots most often studied in humans include two distinct upper body depots on the ventral side, superficial and deep, as well as gluteal and femoral fat pads. Several depots are defined as intraabdominal, including perinephric around the kidneys, retroperitoneal in the retroperitoneal space behind the kidneys and mesenteric and omental WAT lining organs of the digestive tract. (**B**) Several human BAT depots are depicted. BAT in adult humans is present in several different locations, including the neck, heart, spinal cord and kidneys. However, the majority is contained within the supraclavicular depot. (**C**) Four commonly-studied WAT depots in a male mouse are shown. Inguinal fat is a subQ depot that lies just beneath the skin and is similar to the gluteofemoral subQ in humans. Intraabdominal depots include mesenteric fat associated with the intestine, retroperitoneal behind the kidneys (K) and epididymal WAT next to the testes (paraovarian surrounds the ovaries of female mice; collectively, epididymal and paraovarian are also called perigonadal). According to the stricter rules of nomenclature, only mesenteric fat is a true visceral depot in mice, as it is the only one that drains into the portal vein. (**D**) The location of the interscapular BAT depot in mice, the only dissectible BAT fat pad, is shown. Abd sc: Abdominal subcutaneous; IA: Intraabdominal; VISC: Visceral. Figure 1A is adapted with permission from [1]. Copyright 2013 Elsevier. Figure 1B is adapted with permission from [35]. Copyright 2010 Wiley-Blackwell. Figure 1C is adapted with permission from [36,37]. Copyright 2011 Elsevier. Figure 1D is adapted with permission from [38]. Copyright 2017 John Wiley & Son.

Unlike other organs that typically have a single anatomical location, AT is distributed throughout the body in discrete locations referred to as depots. WAT depots can be broadly classified as either subcutaneous (subQ) or intra-abdominal. SubQ WAT is located superficially just beneath the skin and is the predominant location in a normal weight individual, accounting for approximately 80% of all body fat [29,30]. Intraabdominal WAT can be further broken down into visceral or non-visceral and collectively accounts for up to 10–20% of total fat in men and 5–8% in women [29]. Many distinguish intraabdominal as visceral versus non-visceral depots based on whether the blood is drained directly to the liver through the portal vein [31]. With this restrictive definition, only omental and mesenteric are true visceral depots, while the fat pads around the kidney (retroperitoneal and perinephric) and heart (epicardial) would be considered non-visceral. Depots commonly studied in mice include inguinal subQ, mesenteric, retroperitoneal and perigonadal (epididymal in males and paraovarian in females; collectively referred to as perigonadal). Major WAT depots in humans and mice are illustrated in Figure 1A,C.

Since recent evidence demonstrates notable depot-specific differences in WAT, it is important to acknowledge the similarities and distinctions in WAT depots between mice versus men. The inguinal fat pad in mice appears comparable to the large gluteofemoral subcutaneous depot in humans, and the mesenteric fat pad in mice is considered the most analogous to human visceral WAT both in its location and function, because it has access to the portal vein. However, the mesenteric depot is difficult to dissect in mice, resulting in few studies that include this depot in their analyses. Another notable distinction between the two species is the absence of a comparable perigonadal fat pad in humans. This difference is particularly important, as most studies in mice have utilized the perigonadal depot to represent visceral WAT due to its ease of dissection and large absolute mass. However, caution is advised when trying to extrapolate findings from this depot to human visceral WAT function as humans do not have an analogous fat pad.

Like WAT, BAT is also present in several discrete locations throughout the body. In mice and human infants, the most prominent BAT depot is located in the interscapular region (between the shoulder blades) [32]. In humans, interscapular BAT seems to gradually decline with age, although highly variable with temperature as previously noted. Recently, the use of fluorodeoxyglucose positron emission tomography with computed tomography (FDG-PET/CT) scans coupled with tissue biopsies has confirmed the presence of active BAT depots in adult humans at various sites, with the main depot in the supraclavicular region, but also in the neck, para-aortic, paravertebral and suprarenal regions [2,33,34]. Because of the sensitivity of this depot to temperature and because mice require a higher temperature to reach thermoneutrality, housing conditions need to be considered when comparing molecular signatures of this depot between mice and man. Major BAT depots for man and mouse are shown in Figure 1B,D.

There are numerous functional differences between AT depots. These differences contribute to the well-accepted adage that visceral WAT is positively correlated with higher incidence of metabolic disease, inflammation and insulin resistance, serving as an independent risk factor for type 2 diabetes, hypertension and all-cause mortality [39]. On the other hand, subQ WAT deposition does not confer disease risk and may instead be protective [29]. WAT removal and transplantation studies in mice support a detrimental role for visceral WAT and an inherent beneficial role for subQ fat. For example, while surgical removal of mesenteric fat is not feasible due to the heavy innervation and vascularization, removal of epididymal and retroperitoneal fat improves metabolic profile and longevity in rodents [40,41]. Further, improved glucose homeostasis and decreased adiposity are observed when subQ WAT is transplanted into the visceral depot [42]. These depot-specific features can be further modified by factors such as age, sex and race. That is, with advancing age, there is a redistribution of subQ fat to visceral depots, as well as greater ectopic fat deposition [43,44]; women accumulate more subQ fat mass and more total fat mass, while men carry a greater proportion of fat in visceral depots [45]. Furthermore, when comparing WAT distribution in different races, African American individuals have reduced visceral WAT deposition compared to Hispanic and Caucasian populations, while South Asians have relatively greater visceral adiposity compared to Caucasians [46,47]. To add additional complexity, while differences among depots are now well recognized, evidence also exists for regional differences within a single depot. For example, structural and functional heterogeneity within a single inguinal subQ fat pad has been reported with ‘browning’ being more restricted to the core of the depot [48].

The characteristics that vary among depots include adipocyte size, developmental origin, cellular composition, extracellular matrix, innervation, vascularization, metabolism, adipogenic potential and beiging capacity among others. While a thorough review of each is beyond the scope of this review, it is critical to appreciate that these differences result in WAT depots that are structurally and functionally distinct and exhibit marked differences despite all being classified as a single tissue. Thus, it might be expected that each depot would respond uniquely to a hormonal signal, such as growth hormone, as will be discussed in more detail later in this review. Ultimately, one has to use caution when evaluating a single depot or even a small tissue section as representative of AT in general.

### 2.4. Other Cellular and Non-Cellular Components of AT

Adipocytes are not the only cell type in AT; in fact, in some depots, the non-adipocyte cells can outnumber the adipocytes despite adipocytes occupying the largest volume of the tissue [1]. The other cell types are collectively referred to as the stromal vascular fraction (SVF) based on their method of isolation, which includes enzymatic digestion of AT followed by centrifugation. With this standard fractionation method, the adipocytes float, and the pellet contains the remaining cells within the SVF. The SVF contains a variety of immune cells, as well as preadipocytes, endothelial cells, neural cells, senescent cells and fibroblasts. The combined role of these other cell types in the function of AT is an area of significant interest, in part because its composition can vary dramatically among depots, with obesity, environmental changes (temperature) or disease states. For example, immune cell populations are significantly altered by AT depot as greater numbers of macrophages are reported in visceral WAT compared to subQ regardless of body weight [49]. Adipose tissue in lean individuals contains more anti-inflammatory immune cells, such as M2 macrophages, eosinophils, invariant natural killer T (iNKT) cells, Th2 cells and Treg cells. In contrast, AT in obese individuals has a dramatic shift in immune cell populations, with dramatic increases in M1 macrophages, neutrophils, mast cells, Th1 cells and CD8 T cells [50,51]. Other immune cell changes, like increased natural killer cells and decreased T regulatory cells, exacerbate the inflammatory state of the tissue with obesity [50,52]. While the immune cell shift that occurs with obesity is well documented, it is just one example of the dynamic nature of this tissue.

Other cells or structural components of AT that are important to introduce with respect to GH are the senescent cell population along with the extracellular matrix (ECM) composition of the tissue. Cellular senescence is a process by which cells undergo irreversible cell-cycle arrest and is accompanied by distinctive alteration in the cell’s secretome, referred to as senescence-associated secretory phenotype (SASP). Senescent cell accumulation is thought to be triggered by various factors such as oncogene activation, oxidative stress, telomere shortening, DNA lesions, epigenetic alterations or exposure to other metabolic stressors, such as high glucose [53,54]. Senescent cells are present in AT [43], and the accumulation of these cells has a major impact on AT homeostasis irrespective of AT mass [55,56]. Importantly, senescent cell accumulation is implicated in age-related phenotypes, and their removal has been shown to extend health span and lifespan in at least mice [57,58]. Likewise, the ECM, which surrounds individual cells and provides structural support for the tissue, requires extensive and repeated remodeling to accommodate changes in adipocyte size and AT mass [59]. Like senescence, excessive accumulation of ECM, or fibrosis, is linked to AT dysfunction. Collagens are the most abundant ECM component, and collagens V and VI, in particular, appear to have significant impact on the health of AT during tissue expansion and obesity. For example, increased collagen V plays a role in inhibition of angiogenesis [60], and collagen VI appears to be critical in reducing plasticity and limiting uncontrolled expansion of the adipocytes, as well as in the development of fibrosis [61,62]. Differences in senescence and ECM composition between fat depots may provide further explanation of the functional depot differences observed in lean and obese states.

## 3. Clinical Conditions and Mouse Lines with Alterations in GH

**Figure 2:**
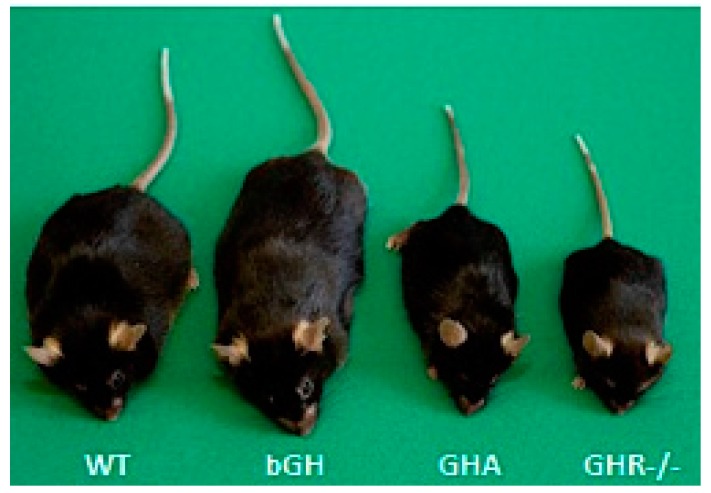
Mice with altered GH action. From left to right: a wild-type mouse, a bGH mouse with increased GH action, a GHA mouse with decreased GH action and a GHR-/- mouse with GH insensitivity. Adapted with permission from [36,37]. Copyright 2011 Elsevier.

To understand the role of GH on AT, it is critical to use in vivo models. While multiple organisms have been utilized, this review will focus on *Mus musculus* or “mice”. Extremes in the GH/IGF-1 axis in both humans and mice have allowed researchers to uncover many of GH’s actions at the tissue level. For example, humans with acromegaly/gigantism and bovine GH transgenic mice (bGH) provide an opportunity to evaluate the role of chronic excess GH action while mice or humans treated with exogenous GH allow for evaluation of acute GH effects. Decreased GH action, as found with GH deficiency (GHD) in humans, can be emulated depending on severity by a number of mouse lines including growth hormone receptor (GHR) antagonist (GHA) mice, mice with an inducible means to reduce GH action in adulthood, or Ames dwarf mice, which are completely GH deficient. Finally, humans with Laron syndrome (LS) and GHR gene disrupted (GHR-/- or aGHRKO) mice, which are fully or partially unable to respond to GH, provide an opportunity to evaluate GH insensitivity. Table 1 summarizes the clinical conditions along with examples of comparable mouse lines; a brief description of each is also provided below. Figure 2 depicts many of the mouse lines described. Importantly, these mouse lines, which share many features with their respective clinical conditions, provide an opportunity to do more invasive analyses of multiple AT depots, making them a valuable tool to study the effects of GH on this tissue. Of note, many other mouse lines exist to explore the physiological and metabolic impact of GH action; however, this review will describe select lines that are most similar to the aforementioned clinical conditions and that have significant data related to AT.

### 3.1. Elevated GH: Acromegaly and bGH Transgenic Mice

Acromegaly with or without gigantism is a disorder characterized by elevated levels of GH and IGF-1. Gigantism occurs when acromegaly starts in childhood prior to growth plate fusion resulting in extreme longitudinal bone growth. Acromegaly is usually caused by an adenoma in the anterior pituitary. However, ectopic tumors outside the pituitary that produce GH or GHRH have also been described [64,65,66]. Since elevated GH blocks insulin action, individuals with acromegaly are prone to hyperglycemia, insulin resistance and type 2 diabetes [67]. They also have a two-fold increase in overall mortality with increased rates of heart disease [67,68,69] and cancer [70,71,72,73]. It is estimated that if left untreated, acromegaly decreases lifespan by ten years [64].

Bovine GH transgenic (bGH) mice have been genetically engineered to constitutively overexpress GH. Note that transgene expression is constitutive and not isolated to the pituitary. Similar to acromegaly, bGH mice have elevated plasma levels of GH and IGF-1 [74]. Overall, these mice are giant and so technically more similar to gigantism than adult onset acromegaly. These mice experience dramatic increases in lean mass, have disrupted glucose homeostasis, insulin resistance and hyperinsulinemia [74,75,76]. bGH mice also develop cardiomegaly, hepatomegaly and have marked cardiac, vascular and kidney damage [77,78,79]. Additionally, bGH mice have a greater incidence of tumors and dramatically shorter lifespans, reduced by approximately 50% compared to their littermate controls [78,80]. Due to these similarities, bGH mice provide an opportunity to more invasively study the condition of acromegaly.

Note that GH injection in genetically normal mice or humans can also reveal the acute effects of GH action. As might be expected, the experimental conditions and the inclusion/exclusion criteria (age, sex, obesity status, etc.) of subjects/animals employed and the GH injection scheme (dose, timing) dramatically influence the results and so will not be discussed here. However, studies that have used acute GH treatment methods will be discussed in the context of AT and obesity management below.

### 3.2. GH Deficiency: GHD and GHA and Ames Dwarf Mice

GHD in children and adults is defined by low levels of GH and IGF-1 [81]. The pathogenesis of GHD differs between congenital and acquired deficiencies. Congenital GHD is caused by either the absence of the pituitary gland or genetic mutations in GH, GHRH or pituitary transcription factors [81]. Acquired GHD arises from different and widely-varying causes, such as non-functioning pituitary adenoma, central nervous system trauma and idiopathic hypopituitarism [82]. The clinical presentation of GHD depends on the age of onset. Children with congenital or acquired GHD exhibit stunted growth and reduced lean mass; they also have a higher risk of developing hyperlipidemia and dramatic hypoglycemic episodes [83]. The lack of growth tends to be more exaggerated for children with congenital GHD than children diagnosed with acquired GHD. Adults with GHD have more generalized and nonspecific symptoms, including decreased muscle mass and energy, lower bone density, anxiety, depression and an overall decline in quality of life [84,85]. Please check and confirm, the highlighted part could be two sentences.

GHR antagonist transgenic (GHA) mice have a number of similarities with congenital GHD. GHA mice express a mutated bGH gene in which the codon for glycine at position 119 is substituted for a larger amino acid [86]. This single amino acid substitution results in the production of a protein that competes with endogenous GH for GHR binding and results in a marked reduction, but not elimination, of GH-induced intracellular signaling. GHA mice are dwarf and intermediate in size between the global GHR knockout (GHR-/-) and wild-type (WT) mice (Figure 2) [78]. GHA mice have reduced levels of IGF-1 and lean mass [75]. They also have normal to slightly improved insulin sensitivity, although there is evidence that their insulin sensitivity deteriorates at older ages [87]. Strikingly, the GHA mice are the only mice that have decreased GH action that do not exhibit lifespan extension [88]; however, only one study to date has been published assessing lifespan in these mice with relatively low numbers, and females did tend to live longer, although this difference did not reach statistical significance.

Ames dwarf mice have a mutation in the *Prop-1* gene resulting in multiple pituitary hormone deficiencies (GH, prolactin and thyroid-stimulating hormone) [89]. Thus, they are similar to a congenital GH-deficient state. Although they have multiple hormone deficiencies, they share many features with GHR-/- mice (discussed below), have extreme insulin sensitivity and increases in lifespan. Yet there are differences between GHR-/- and Ames dwarf mice. For example, calorie restriction does not further extend lifespan in GHR-/- mice, while it does in Ames dwarf mice [90,91]. Phenotypically similar to Ames dwarf mice, Snell dwarf mice have multiple pituitary hormone deficiencies due to a mutation of the *Pit-1* gene [92]. While Snell dwarf mice have been extensively characterized with respect to aging and specific tissue alterations, few studies have evaluated the AT in these mice [93]. Another mouse line that has a more targeted approach to disrupt just the somatotrophs in adulthood and that more closely resembles adult onset GHD was recently described [94]. The adult onset-isolated GHD line (AOiGHD) has the ablation of somatotrophs with an inducible Cre-Lox system that utilizes the diphtheria toxin gene [94]. Using an induction time starting at 10–12 weeks of age, circulating levels of GH and IGF-1 are decreased, but still detectable in these mice. Interestingly, even partial GH disruption, as shown in these mice, has a dramatic impact on metabolic function, resulting in improved insulin sensitivity.

### 3.3. GH Insensitivity: Laron Syndrome, GHR-/- Mice and aGHRKO Mice

Laron syndrome (LS) is a genetic disorder characterized by GH insensitivity. An autosomal recessive mutation in the *GHR* gene has been identified as the most common cause of LS. This mutation renders the GHR protein non-functional, effectively preventing downstream actions of GH, including the production of IGF-1 [95]. Due to perturbations in the negative feedback from IGF-1, LS is accompanied by elevated levels of circulation GH [95], but severe growth retardation and reduced lean body mass [96]. Thus, while individuals with LS present similar growth retardation patterns as patients with congenital GHD, LS patients are more challenging to treat since they are completely resistant to GH therapy. The incidence of insulin resistance and type 2 diabetes varies among the different LS cohorts. Notably, Israeli and Turkish cohorts are more likely to have hyperinsulinemia and can develop type 2 diabetes later in life [96,97], while Ecuadorian cohorts have improved measures of glucose homeostasis, including improved insulin sensitivity and resistance to type 2 diabetes [98]. Interestingly, all cohorts appear to be protected from cancer [96,99,100,101].

The GHR-/- mice are analogous to LS patients [102]. These mice are resistant to GH action with very low IGF-1 and elevated GH levels [103]. GHR-/- mice are notably dwarf, about half the size of littermate controls (Figure 2), and have decreased lean mass along with smaller organs [75,104]. These mice are extremely insulin sensitive [75,105] and are resistant to many age-associated complications compared to their littermate controls. In contrast to what is observed in bGH mice, GHR-/- mice are protected from neoplastic diseases [106]. As might be expected, GHR-/- mice are extremely long-lived, living about a year longer than a control mouse. This increased lifespan has been reproduced in different laboratories and under different experimental conditions, including alterations in sex, genetic background and diet composition [88,91,107]. In addition to congenital global disruption, a second line, called aGHRKO for adult onset GHR deletion, was developed in which the *Ghr* is temporally deleted using a ubiquitously-expressed tamoxifen-inducible Cre-Lox system [108]. Induction in these mice occurred at six weeks of age. While the Cre recombinase gene used in this study was very effective for removal of GHR from liver, its removal from many other tissues was partial or incomplete. As such, some, but diminished, GH signaling remains in most extrahepatic tissues. These mice have improved insulin sensitivity, decreased IGF-1 and body weight and an increase in maximal lifespan for at least the female mice [108].

## 4. Adipose Tissue and GH

As will be described below, GH has a dramatic impact on AT. GH levels in circulation are inversely related with AT mass in both humans and mice, and as might be expected, these changes result in significant shifts in adipokine secretion patterns. The action of GH on AT provides a unique perspective on AT quality versus quantity. That is, since GH reduces AT mass, one would expect that GH’s impact on this tissue would be positive as reduced AT mass is usually considered favorable for overall health. However, there is mounting evidence that despite a reduction in AT mass caused by GH, the metabolic health or “quality” of the tissue is compromised. This is most apparent in mice as more invasive measures are feasible. That is, mice with excess GH action, such as bGH mice, are lean, but characteristics of the AT might be considered deleterious and are accompanied by negative consequences on overall metabolism and lifespan. Conversely, mice with a reduction in GH action are relatively obese, yet are more insulin sensitive and do not have the metabolic perturbations that accompany obese states. Thus, GH in excess appears to create a counterintuitive unhealthy lean state, while reductions in GH promote a healthy obese state. This disconnect between quantity and quality provides an intriguing means to evaluate not only how GH impacts AT, but to better understand the characteristics of AT that are responsible for metabolic dysfunction in obesity.

### 4.1. Body Composition

**Figure 3:**
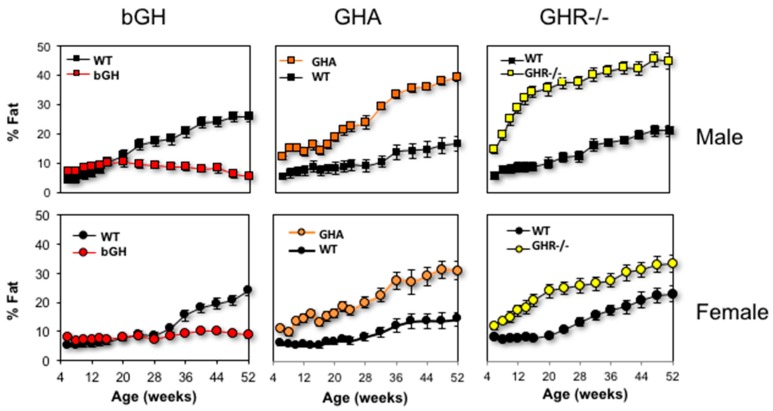
Comparison of body fat percentage in mice with altered GH action. Male and female bGH mice have greater body fat percent than WT mice earlier in life, a trend that starts to reverse at four and six months of age, respectively (left). Fat percentage is greater in male and female GHA mice compared to controls throughout life. Male GHR-/- mice have markedly increased body fat percent compared to controls and appear to rapidly accumulate fat during the first four months of life. Increased percentage of fat is also observed in female GHR-/- mice compared to controls, albeit not as drastic. Adapted with permission from [38]. Copyright 2017 John Wiley & Son.

With its potent lipolytic effect, one would expect total body fat to be drastically reduced with excess GH. Indeed, acromegaly results in reduced total body fat [75,109,110], and treatment of acromegaly to normalize GH levels increases body fat [111,112]. Similarly, adult bGH mice have less total body fat than littermate controls (Figure 3) [75]. However, bGH mice have greater fat mass at younger ages (less than three months of age for males and four months of age in females) [76,113,114]. Longitudinal body composition studies reveal several other intriguing trends. First, the absolute fat mass of bGH mice remains relatively consistent throughout the first year of life; in fact, in this study, there is no significant difference in the fat mass between the first body composition measurement made at six weeks compared with the final measurement at 52 weeks [76]. Thus, bGH mice are resistant to any significant AT accumulation. Additionally, bGH mice fed HF diets are also resistant to diet-induced obesity, exhibiting preferential accumulation of lean tissue instead of AT [77,115]. Second, sex-specific differences are apparent with bGH females showing some delay and less exaggerated changes in body composition as compared to the bGH males. From an acute perspective, treatment of obese C57Bl/6J mice with exogenous GH dramatically reduces fat mass in a dose-dependent manner [116]. Collectively, these results would suggest that age and sex are important parameters to consider when interpreting the relationship of GH to body composition.

At the opposite end of the spectrum, decreased GH action consistently increases adiposity. GHD in children and adults causes increased fat mass [117,118], which somewhat reverses when treated with recombinant human GH (rhGH) [119]. Patients with LS also experience increased central and total adiposity [96]. Mice show a similar trend. GHA, GHR-/-, AOiGHD, aGHRKO and Ames dwarf mice have increased adiposity, which like bGH mice appears to be sex specific with the increase in AT less dramatic for females [87,94,104,108,120,121,122]. Further, when AOiGHD, Ames, GHA and GHR-/- mice are fed a HFD, all lines are more susceptible to gaining additional fat mass when compared to WT mice and, yet, remain resilient to the detrimental metabolic effects of high fat feeding on glucose homeostasis and insulin sensitivity [77,94,121,123,124]. Thus, overall the relationship of GH to total adiposity is inverse and fairly consistent from mouse to man.

### 4.2. Depot Specific Differences

Mouse studies provide strong evidence that GH affects AT in a depot-specific manner. While clinical studies support this notion, the impact on a specific depot is not always consistent from mouse to man. While the inconsistency could be due to inherent differences in depots between man and mouse as already discussed, it is also likely that the available methodologies for humans to sample and study various depots is limited versus what can be done in mice. Regardless, depot-specific differences have been reported in humans. In acromegaly, all depots appear to be decreased, but the greatest reduction occurs in the visceral depots [125]. For example, one study with adults with GHD treated with GH (0.013–0.026 mg/kg/d) for 26 weeks reports a total fat mass reduction of 9.4%, with again the visceral AT being more dramatically reduced (30% decreased) versus the subQ AT (13% reduction) [126]. However, there is also clinical evidence for GH preferentially impacting subQ depots. In LS, while there are marked increases in AT in both subQ and intra-abdominal regions, a larger percentage of AT is distributed in the subcutaneous region of the limbs [63]. Clearly, data from humans suggest a depot-specific role for GH on fat mass; however, the ability to directly compare depots from the same clinical sample is challenging.

**Figure 4:**
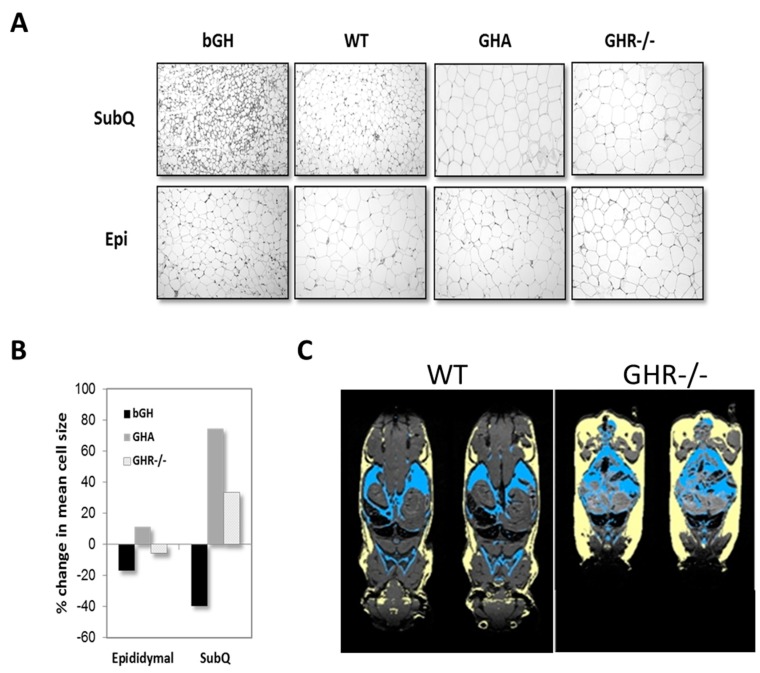
Depot-specific differences due to extremes in GH. (**A**) Hematoxylin and eosin staining of subcutaneous (SubQ) and epididymal (Epi) AT. Tissue samples were obtained from six-month-old GHR-/-, GHA, bGH and control mice. (**B**) Quantification of adipocyte size from these mice. (**C**) Adiposity in GHR-/- mice. Regional body fat distribution of male WT mice (left) and male GHR-/- mice (right) using magnetic resonance imaging (MRI). The mouse is positioned with the anterior part at the bottom of the image. Subcutaneous AT is highlighted yellow and intra-abdominal blue. Adapted with permission from [36,37]. Copyright 2011 Elsevier.

**Figure 5:**
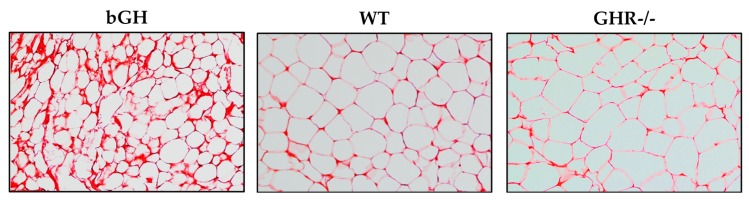
Comparison collagen staining in AT. SubQ AT from five-month-old bGH, wild-type (WT) controls and GHR-/- mice stained with Picrosirius red, a commonly-used histological technique to visualize collagen in paraffin-embedded tissue sections. Adapted with permission from [38]. Copyright 2017 John Wiley & Son.

Because multiple depots can be sampled from a single animal, much of what we understand about GH’s impact on specific depots has come from studies using mice. One of the most striking observations reported has been a preferential enlargement of the inguinal subQ depot in mice with reduced GH action. This finding has been fairly consistent and reported in male and female GHA, GHR-/-, aGHRKO and Ames dwarf mice [75,87,104,108]. With an excess in GH action as in bGH mice, all depots appear to be similarly reduced; however, molecular characterization of the AT depots reveals a more significant impact in subQ depots as compared to others [127]. GH treatment (four distinct doses for six weeks) in obese, male C57BL/6J mice reveals that the inguinal subQ and mesenteric depots are most sensitive to acute GH treatment and in a dose-dependent manner [116]. The targeted impact of GH on subQ AT is readily apparent when comparing AT histology among depots. As shown in Figure 4A,B, hematoxylin and eosin-stained tissue sections from mouse lines with extremes in GH action show dramatic alterations in morphology and adipocyte size in subQ AT; yet, epididymal AT is more uniform despite extreme differences in GH signaling. Figure 4C provides an image showing the dramatic increase in all subQ depots of GHR-/- mice relative to controls. Although not designed to compare depots, surgical removal studies of epididymal and perirenal depots of both GHR-/- and Ames dwarf mice, which have AT that develops in the absence of GH signaling, show that these depots have distinct beneficial effects as compared to these same depots in control mice [128,129]. Likewise, transplantation studies with these depots from GHR-/- mice into control mice further highlight an inherent beneficial role of these other non-subQ AT depots [130]. Thus, although the epididymal depot may not appear to be different histologically (Figure 5) or by some of the other variables measured to date, there does appear to be a unique and inherent benefit to these non-subQ AT depots when they develop in the absence of GH signaling. Many other examples in the literature support depot-specific differences at the cellular or molecular level due to GH action. Although not an exhaustive list, some recent examples of depot-specific differences are provided in Table 2.

### 4.3. Cellular and Non-Cellular Components Altered by GH

GH and/or IGF-1 have frequently been implicated as important hormones in the proliferation and differentiation of preadipocytes. However, the findings from these studies have been somewhat inconsistent. The variability in findings appears to be influenced by whether isolated primary or clonal cells are used [136], and for primary cells, results differ depending on the depot used for isolation of the primary cells. For example, proliferation and differentiation are suppressed in preadipocytes isolated from paraovarian depots in GHR-/- mice, whereas preadipocytes isolated from the subQ depot of GHR-/- mice behave similarly to those isolated from the same depot from control mice [137]. Using cell sorting methodology to ensure isolation of a pure cell population, subQ mesenchymal stem cells from GHR-/- and bGH mice exhibit increased and decreased adipogenesis, respectively, as well as a role for the Wnt/β-catenin signaling pathway as important for controlling adipogenesis in these mice [135].

GH has been implicated in AT cellular senescence in mice. Senescent cell accumulation increases in the subQ depot and mesenteric depots of female GH-injected mice and all depots except paraovarian in female bGH mice [133]. Conversely, less accumulation of senescent cells is seen in most depots of Ames and GHR-/- mice (see Table 2 for specific depots). As accumulation of senescent cells has been linked to aging, it is curious that GHA mice that do not appear to have an increase in lifespan also have comparable levels of WAT senescence as control animals [138]. This observation provides further support for a role of cellular senescence in lifespan.

Few studies have examined how GH alters AT immune cell populations or their activation state, and all published studies have utilized only AT samples collected from mice. bGH mice exhibit depot-dependent alterations in immune cell populations [127] with greater M2 macrophage and regulatory T cell infiltration in subQ and mesenteric AT depots and with few significant changes in the epididymal depot. In this same study, RNA-seq analyses reveal that the dramatically altered pathways in bGH subQ AT are T cell infiltration and its activation pathways, which also are not significantly altered in the bGH epididymal depot. Conversely, using GHR-/- mice and epididymal-derived macrophages, NLrp3 inflammasome-mediated inflammation, which plays a central role in the inflammatory response and in diverse human diseases, is reduced, suggesting a role for GH in macrophage inflammation [139]. Furthermore, IL-6 levels in epididymal and retroperitoneal AT of GHR-/- mice are reduced compared to controls [128]. Unfortunately, these studies did not evaluate other AT depots such as mesenteric and subQ. Regardless, the absence of GH action seems to shift at least epididymal AT from a pro-inflammatory to anti-inflammatory state. Current research has also suggested the presence of other immune cells in AT, such as eosinophils, natural killer and mast cells, although the role of GH on these cells populations has yet to be investigated. While there are many unanswered questions remaining, these studies do provide strong evidence for GH influencing the AT immune cell populations.

GH, with its well-documented role to modify the ECM in other tissues [140,141], is likely able to influence the ECM in AT. Evidence from clinical and mouse data on the relationship between GH and WAT ECM is minimal. Microarray analysis of isolated subQ adipocytes from GHD patients after GH treatment shows differential expression of several genes encoding components of the ECM, such as collagen family member COL4A6 and laminin α 3 [142]. Using mice with altered GH, our laboratory has compelling data that GH action is positively correlated with ECM deposition [143]. Specifically, bGH mice have increased AT collagen staining and hydroxyproline content, whereas GHA mice and other mice with a reduction in GH action have a decrease in both. Moreover, collagen deposition is not uniform among the depots, with subQ most effected and with regional differences within a depot (pockets of significant fibrosis). While these data in mice are compelling, they do not show whether the role of GH in ECM deposition is a direct effect of GH or an indirect consequence of the increase in lipolysis or the other hormonal alterations in these mice.

### 4.4. Brown Adipose Tissue and Beige Adipose Tissue

GH has been implicated to have an influence on BAT, as well as WAT beiging with, again, these data coming from mice. Studies have been somewhat conflicting, although this may relate to differences in tissue isolation or temperature at which the mice are housed. GHA, Ames dwarf and GHR-/- mice have been reported to have enlarged BAT depots, as well as increased UCP1 RNA expression [144,145]. BAT weight and UCP1 expression in bGH mice have been shown in some studies to be similar to controls and in other studies to be increased in size with greater UCP1 expression [115,145]. Similar to the latter study, acute GH treatment (1 mg/kg/day) of genetically-obese mice does promote increases in UCP1, UCP2 and UCP3 expression, although changes in BAT mass are only observed with a higher GH dose (3.5 mg/kg/day) [146]. BAT samples from GHR-/- mice and controls have been evaluated in a whole-genome microarray studies and show increases in the expression of a number of metabolism-associated genes in BAT from the GHR-/- mice, as well as decreases in pro-inflammatory gene expression compared to controls [134]. Recently, Darcy et al. [144] report the impact of BAT removal in Ames dwarf mice. Removal of the intrascapular BAT depot was shown to be critical for energy metabolism and thermogenesis in the Ames dwarf mice relative to their littermate controls. Interestingly, BAT removal in Ames dwarf, but not control mice caused an increase in the use of lipids from WAT, which decreased the mass and adipocyte size in only Ames dwarf mice. The authors explain this as an increase in energy demand for thermogenesis relying more on the lipid from WAT when BAT is absent. Thus, GH likely influences BAT function, although more targeted studies are needed. The role of GH on WAT beiging remains to be elucidated.

### 4.5. Adipokines

Adipokine levels are altered in humans and mice with extremes in GH-induced signaling. Leptin and adiponectin are the two adipokines most commonly studied. Overall, GH action is negatively correlated with both leptin and adiponectin levels. For example, individuals with acromegaly and bGH mice have decreased levels of leptin and adiponectin, while treatment increases at least leptin levels [105,147,148,149,150]. While data from GHD patients vary based on age and likely etiology, adipokine levels in LS and mouse lines with reduced GH action are fairly consistent and show an opposite trend to that of acromegaly and bGH mice. Patients with LS have two- to three-fold higher total and high molecular weight (HMW) adiponectin levels as compared to subjects of corresponding age, gender and degree of adiposity [148,151]. Interestingly, leptin levels are elevated in LS as compared to lean, age- and gender-matched controls, but not different from obesity-matched controls, suggesting LS that leptin is more closely tied to AT mass [152]. Ames, GHA, GHR-/-, AOiGHD and aGHRKO mice have either unchanged or elevated leptin levels, which is partly dependent on age, sex and fat mass accumulation, while total and HMW adiponectin levels are elevated in all of these lines [75,94,105,108,151]. It is worth noting that these two adipokines, leptin and adiponectin, show an interesting relationship with fat mass and GH signaling. That is, adiponectin and leptin follow the same pattern when in many systems, they are oppositely regulated. Although leptin levels are consistent with what would be expected based on fat mass, low levels of leptin rather than high levels of leptin are more commonly associated with improved insulin sensitivity, an improved metabolic profile and increases in longevity [153,154]. Adiponectin is usually negatively associated with obesity and positively associated with insulin sensitivity and longevity [153,154]. In these mice, as well as the comparable clinical conditions, adiponectin is positively associated with insulin sensitivity and longevity, as might be expected, but also with fat mass. This uncommon association has given substantial attention to these adipokines and their role in promoting the GH-induced phenotype.

Other adipokines have been assessed in a few studies, but these reports have been sporadic, making it difficult to draw major conclusions. Of the adipokines studied, vaspin, visfatin and omentin are increased in acromegaly and decreased after normalization of GH levels [135,155]. In fact, vaspin and visfatin, which are adipokines associated with visceral AT mass, have been proposed as biomarkers of visceral AT dysfunction with acromegaly [155]. Resistin levels have been evaluated in several lines and clinical conditions, but the trend is highly variable with decreased levels in bGH mice and no alterations or reduced resistin levels in GHD [156,157]. Circulating FGF21 levels have been shown to be elevated in bGH mice, but unchanged in GHR-/- mice [158].

A summary of the known changes in AT with GH in the mouse lines in provided in Table 3 below.

## 5. Does GH or GH Antagonist Have Potential for Treatment of Obesity/Lipodystrophy?

### 5.1. Obesity

While treatment of obesity should start with comprehensive changes in lifestyle, pharmaceutical methods for obesity management are still desperately needed. There are several reasons why GH might be considered a viable therapeutic option. First, GH has a potent ability to reduce fat mass, as already described, but also can preserve lean mass; the preservation of lean mass is not typically achieved by many other methods for weight loss. Second, obesity, especially visceral obesity, can be considered a state of relative GH deficiency. That is, human visceral obesity is negatively correlated with spontaneous and stimulated GH secretion [159], and substantial weight loss restores GH secretion patterns [160].

Numerous studies have evaluated the efficacy of using GH for obesity management, and these studies have been recently summarized [161,162]. There are many confounding factors in these studies—GH dose, inclusion/exclusion criteria, sample size, method of assessing AT mass, length of follow up and timing of glucose and insulin measurements relative to GH dose—that need to be considered in their collective interpretation. Overall, GH therapy results in the reduction of total and visceral AT in clinical trials [163,164,165,166,167,168]. Further, studies that simultaneously restrict calories show the added benefit of attenuating the loss of lean body mass with GH therapy [169,170,171,172]. However, a meta-analysis of 24 clinical studies on the use of GH therapy for obesity concluded that the effects on body composition and lipid profiles are modest and do not reduce weight sufficiently to be considered beneficial [173]. Of concern, adverse side effects—fluid retention and carpal tunnel syndrome, as well as deterioration in glucose metabolism—have been reported in some [165,168,171,174,175], but not all [163,167], studies. Interestingly, several studies have evaluated GH treatment in obese individuals with diabetes and showed that GH treatment not only reduced visceral AT mass and levels of LDL cholesterol, but also improved insulin sensitivity [176,177]; thus, the loss in fat mass may be more influential than the diabetogenic actions of the GH treatment. Studies in mice would support this, as diet-induced obese, diabetic mice treated with GH have significant weight loss and improvements in glucose metabolism [116]. Overall, while GH has great potential for treating obesity, its cost, the modest impact, the concerns related to side effects and the lack of larger and longer controlled trials makes it unlikely that GH will be of therapeutic value for obesity in the near future.

Note that AT mass may not be the best clinical readout for GH’s impact on obesity. In fact, the data presented here from mice would suggest that GH may be very effective at reducing AT mass, but may chronically increase several hallmarks of AT dysfunction (increase in cellular senescence and fibrosis, as well as the promotion of an inflammatory immune cell profile). Therefore, an intriguing question is whether a GH antagonist may have benefit in the management of obesity. After all, the obese GHA and aGHRKO mice are metabolically healthy. Thus, GHA treatment would not be expected to reduce fat mass and therefore remedy obesity, yet it may disconnect obesity from the unfavorable AT changes and metabolic outcomes that are typically associated with obesity. While the ideal would be to have a therapy that reduces AT mass and simultaneously leaves AT in a healthier state, current research that provides new insights into the AT and the obesity phenotype will continue to evolve and refine our understanding of how the GH/IGF axis alters this tissue. Importantly, the reduction in quantity of AT may not be the ultimate goal.

### 5.2. Lipodystrophy

At the opposite end of the adiposity spectrum is lipodystrophy, which refers to a group of heterogeneous disorders characterized by loss of AT. Lipodystrophy can affect the whole body (generalized) or select regions (partial) and can be congenital or acquired. While many forms of lipodystrophy have been identified, one that is relatively common is an acquired form that occurs in an estimated 40%–70% of patients treated with highly active antiretroviral therapy (HAART) for human immunodeficiency virus (HIV) [183]. This form of lipodystrophy is characterized by excess accumulation of visceral fat, peripheral fat atrophy, dyslipidemia and insulin resistance. Important for this review, GH deficiency occurs in approximately one third of the individuals with HIV-related lipodystrophy [184], and GH therapy remains a promising treatment. That is, studies with high dose GH report significant reduction in truncal obesity and improvements in AT atrophy of the limbs [185,186]. However, these studies also reveal major side effects of the high GH dose, including glucose intolerance and diabetes. Low GH doses have been studied with less dramatic impact on AT, but as might be expected, less adverse side effects [186,187].

## 6. Gaps in Knowledge and Conclusions

There are clearly gaps in knowledge with respect to GH and AT. In humans, few studies have included measurements beyond AT mass, leaving many questions unresolved related to the quality of the tissue. Evaluating or sampling of multiple depots is rare and challenging in clinical studies, but important based on our findings from mice. In particular, a better understanding of how GH alters the adipokine secretion, cellular senescence, immune cell profile, beiging and fibrosis is worthy of further exploration in humans. Other recent research on the complexity and dynamic nature of AT has yet to be explored fully in either human or mice, such as angiogenesis, lipid droplet formation and control, and the heterogeneity of the brown, beige and white adipocyte precursor cells. Because of the striking impact of GH on AT and because GH has been implicated in other fields of intense investigation, such as glucose homeostasis and aging, this is a fertile and exciting area of research.

## Figures and Tables

**Table 1:** Phenotypic summary of GH clinical conditions and comparable mouse ones.

	Elevated GH		GH deficiency (GHD)				GH insensitivity		
	Clinical	Mouse	Clinical	Mouse	Mouse	Mouse	Clinical	Mouse	Mouse
	Acromegaly Gigantism	bGH	GHD *	GHA **	AOiGHD	Ames	Laron	aGHRKO ****	GHR-/-
GH defect	Hypersecretion of GH commonly due to pituitary adenoma	Transgenic for bovine GH	Many variations depending on age and etiology	Transgenic for GHR antagonist gene	Ablation of somatotrophs with an inducible system	Mutation in *Prop1*	Hereditary conditions usually caused by GHR receptor defects	Knockdown of *Ghr* gene via an inducible system	Disruption of *Ghr* gene
GH action	↑↑ with onset of adenoma	↑↑ from birth	↓ onset varies based on etiology	↓ throughout life due to GH antagonism	↓ beginning at time of induction (10–12 weeks)	GH deficiency (as well as prolactin and TSH)	Absent from birth	↓ beginning at time of induction (6 weeks)	Absent GHR from birth
GH	↑↑	↑↑	↓	↑		↓	↑	↑	↑
IGF-1	↑↑	↑↑	↓	↓		↓↓	↓↓	↓	↓↓
Growth and body weight	↑↑ *	↑↑	↓ ↔ *	↓	↔	↓↓	↓↓	↓	↓↓
Insulin sensitivity	↓	↓	↑	↑	↓↔	↑	↑↓ ***	↓	↑
Lifespan	↓	↓	↔	No data	↔	↑↑	↔	↔ male; ↑ female	↑

↑ increase, ↔ no change, ↓ decrease; * depends on the age of onset; ** while technically not GH deficient, this mouse line is unique in that it produces a GHR antagonist that blocks endogenous GH, resulting in mice with a dramatic reduction in GH action from birth; *** depends on Israeli or Ecuadorian cohort; Israeli cohort tends to have higher insulin levels [63]; **** GHR disruption is not equivalent in all tissues. Adapted with permission from [38]. Copyright 2017 John Wiley & Son.

**Table 2:** Evidence for depot-specific differences in mice due to extremes in GH action. AoiGHD, adult onset-isolated GHD line.

Model System	Research Focus	Findings	Citation
GHR-/- mice	Proliferation and differentiation of preadipocytes	SubQ derived preadipocytes proliferate, differentiate and respond to hormones in a similar manner to controls Perigonadal preadipocytes from GHR-/- mice fail to differentiate and proliferate normally	[131]
GHR-/- mice	CideA RNA expression	↓ cell-death-inducing DFF45-like effector-A (CideA) levels in subQ AT No difference in CideA expression retroperitoneal or epididymal	[132]
GHR-/-	Proteomic analysis of depot differences with age	Lower levels of Glut4 protein in subQ AT of GHR-/- mice, no difference in epididymal AT Retroperitoneal depot particularly affected by GHR deletion and age	[133]
bGH, GHA, GHR-/-, AoiGHD, Ames	Adiponectin expression	Circulating adiponectin levels correlated strongly with subQ fat mass Higher adiponectin levels in subQ AT of GHR-/- mice	[105]
bGH mice	Immune cell infiltration in AT; RNA-seq analyses of depots	↑ immune cell infiltration (macrophage, T cells) mainly in subQ and mesenteric depots with little change in epididymal AT ↑ in gene expression pathways related to T cell infiltration/activation in subQ, but not epididymal AT	[127]
bGH mice, Snell, Ames, GHR-/- and GH injected mice	Cellular senescence in AT	bGH females: ↑ cellular senescence in all depots except periovarian GH injected WT females: ↑ cellular senescence in subscapular and mesenteric depots GHR-/- females: ↓ cellular senescence all depots except mesenteric Ames: ↓ cellular senescence in paraovarian, mesenteric and subQ	[134]
GHR-/-	Depot whole-genome microarrays	Gene expression differences in gene expression related to metabolic function and inflammation among epididymal, subQ, retroperitoneal AT	[135]
GHR-/-, bGH	AT-derived mesenchymal stem cells	Increased differentiation in cells isolated from subcutaneous AT vs. epididymal	[136]

**Table 3:** Effects of GH on AT in various mouse lines with altered GH action.

	bGH	GHA	AOiGHD	Ames	aGHRKO	GHR-/-
GH Defect	Transgenic for bovine GH	GHR antagonist gene	Adult GH deficiency	Homozygous recessive mutation in *Prop1* (Ames)	Adult induction of GHR deletion	Disruption of GHR gene
WAT						
Mass	↑ young ↓ old	↑↑	↑ (after induction)	↑	↑ (after induction)	↑↑
Depot mass differences	All depots	↑ subQ	↑ subQ/Retro	↑ subQ	↑ subQ; ↑ Epi for males only	↑ subQ
Diet-induced obesity	resistant	Increased susceptibility; impairment in glucose homeostasis with advancing age	Increased susceptibility; preservation of improved glucose homoeostasis	Increased susceptibility; preservation of improved glucose homoeostasis	ND	Increased susceptibility; preservation of improved glucose homoeostasis
Adipokines						
Leptin	↓	↑↑	↑	↑/↔	↑	↑/↔
Adiponectin	↓	↑	↔	↑	↑	↑↑
Resistin	↓	↑	ND	↔	↑ in females	↑
Senescence	↑	↔	ND	ND	ND	↓
Immune Cells **	↑ macrophage, T cells	ND	ND	ND	ND	↓ macrophage inflammation
BAT						
Mass	↑ ↔ ***	↑	ND	↑	↑ in females	↑
UCP1 content	↑	↑	ND	↑	ND	↑↓ ***
References	[75,76,77,78,79,178]	[75,87,105,124,179]	[94,105]	[105,121,180,181]	[108]	[75,77,104,105,145,182]

↑ increase, ↔ no change, ↓ decrease, ND no data. * Measured only in AT. ** Data obtained from flow cytometry only. *** Conflicting data.
